# Draft Genome Sequence of Zoonotic *Streptococcus canis* Isolate G361

**DOI:** 10.1128/genomeA.00967-17

**Published:** 2017-09-21

**Authors:** Inga Eichhorn, Mark van der Linden, Michael Jarek, Marcus Fulde

**Affiliations:** a Institute of Microbiology and Epizootics, Centre for Infection Medicine, Freie Universität Berlin, Berlin, Germany; b Institute of Medical Microbiology and National Reference Center for Streptococci, University Hospital RWTH Aachen, Aachen, Germany; c Genome Analytics, Helmholtz Centre for Infection Research, Braunschweig, Germany; d Department of Medical Microbiology, Helmholtz Centre for Infection Research, Braunschweig, Germany

## Abstract

Here, we report the draft genome sequence of an SCM-positive *Streptococcus canis* strain, G361, isolated from a vaginal swab of a 40-year-old woman. The draft genome comprises 2,045,931 bp in 62 contigs.

## GENOME ANNOUNCEMENT

Although severe infections with beta-hemolytic streptococci in dogs have been known since the late 1930s, *Streptococcus canis* was first described taxonomically in 1986 (1). *S. canis* is considered to be a facultative pathogen, which is normally found to colonize the mucosal surfaces and the skin of its primary hosts, dogs and cats, but without causing disease (2). Under immunosuppressive conditions and stress, *S. canis* is capable of establishing infections ranging from self-limiting alterations of the skin to severe and life-threatening diseases, such as septicemia, meningitis, streptococcal toxic shock-like syndrome, and necrotizing fasciitis (3–6). Notably, *S. canis* has also been classified as an emerging zoonotic agent associated with ulcers, endocarditis, and septicemia in humans both with and without prior dog contact (7–10). However, the number of bacterial isolates from cases of humans suffering from *S. canis* infections remains low in Germany.

Knowledge about the pathogenesis of *S. canis* is scant and relies mostly on comparative phenotypic and genotypic studies to the well-known and closely related human-pathogenic *Streptococcus pyogenes*. Recently, we described the *S. canis* M protein SCM as an important virulence factor that facilitates antiphagocytic activity by plasminogen and nonopsonic IgG binding (11, 12). In addition, *S. canis* is able to immobilize the host’s broad-spectrum serine protease plasmin at the bacterial surface that allows degradation of semisynthetic fibrin thrombi, a pathogenesis mechanism to disseminate and spread into the host (13).

Here, we present the first draft genome sequence of an *S. canis* strain (G361) isolated from humans. The patient was a 40-year-old female from Lower Saxony, Germany, who suffered a premature membrane rupture during pregnancy. The bacterial strain was obtained from a vaginal swab and stored at the German National Reference Center for Streptococci, University Hospital RWTH Aachen.

The 75-bp paired-end reads were generated using an Illumina Genome Analyzer IIx sequencer. The reads were *de novo* assembled into contigs with a minimum size of 500 bp using SPAdes (14). A total of 62 contigs were generated ranging from 630 bp to 191,491 bp, resulting in a total genome size of 2,045,931 bp. The cumulative G+C content of the genome assembly was 39.6%. Gene annotation was performed using the Rapid Annotations using Subsystems Technology (RAST) annotation server (15), which predicted 2,028 coding DNA sequences, 36 tRNAs, and 3 rRNAs in the draft genome. The typical streptococcal virulence-associated genes encoding, e.g., a fibronectin-binding protein, proteins involved in streptolysin S biosynthesis, the previously mentioned SCM protein (antiphagocytic M protein), and an IgG-endopeptidase-related protein were identified in the G361 draft genome. G361 was assigned to sequence type 13 (ST-13) using the multilocus sequence typing service for total-genome-sequenced bacteria from the Center for Genomic Epidemiology (16). No acquired antimicrobial resistance genes were identified within the genome sequences using ResFinder (17). This confirmed the results obtained with the bioMérieux Vitek 2 system, which did not identify any phenotypic resistances to ampicillin, benzyl-penicillin, cefotaxime, ceftriaxone, clindamycin, erythromycin, levofloxacin, linezolid, tetracycline, trimethoprim-sulfamethoxazole, or vancomycin.

### Accession number(s).

This whole-genome shotgun project has been deposited at DDBJ/ENA/GenBank under the accession number NMRV00000000. The version described in this paper is the first version, NMRV01000000.
